# Genetic Basis of Divergent Growth and Muscle Development in Purebred and Crossbred Leizhou Black Goats Revealed by Whole-Genome Resequencing

**DOI:** 10.3390/biology15131038

**Published:** 2026-06-29

**Authors:** Xiaotao Han, Jing Huang, Wenxi Qian, Yuelang Zhang, Ke Wang, Jiancheng Han

**Affiliations:** 1 Zhanjiang Experimental Station, Chinese Academy of Tropical Agricultural Sciences, Zhanjiang 524013, China; xthan0521@163.com; 2 College of Animal Science and Technology, Huazhong Agricultural University, Wuhan 430070, China; 3 College of Animal Science and Technology, Guangxi University, Nanning 530004, China; huangjing07182024@163.com; 4 College of Animal Science and Technology, Tarim University, Alar 843300, China; qianwenxizj@163.com; 5 Hainan Institute, Zhejiang University, Sanya 572024, China; zhangyuelang@zju.edu.cn

**Keywords:** Leizhou black goat, crossbreeding, whole-genome resequencing, muscle development, candidate genes

## Abstract

The Leizhou black goat is a prized indigenous breed in southern China, renowned for its superior meat quality. However, its productivity is constrained by a relatively small body size. Understanding the genetic basis of growth differences between purebred and crossbred populations is essential for designing effective conservation and breeding strategies. To this end, we conducted comparative whole-genome resequencing on 22 purebred (WL) and 50 crossbred (JN: Nubian × Leizhou) goats. Population structure was assessed via principal component analysis, and genomic regions exhibiting elevated genetic differentiation were identified using the population differentiation index (*F*_ST_). Functional enrichment analyses were subsequently applied to genes within these regions. Our results revealed clear genetic differentiation between the two populations. Among the highly differentiated genomic regions, we identified several genes, such as *MYOM2*, *TMTC4*, *DPP6*, and *MIDN*, whose known functions in neural signaling and muscle development make them plausible candidates for contributing to the growth differences between populations. A particularly notable discovery was a non-coding intronic mutation in the RNGTT gene (rs646826802), which is fixed in the paternal Nubian line and prevalent across 25 other global goat breeds but is completely absent in the purebred Leizhou black goat. This study identifies key genetic components associated with trait variation in crossbred goats and reports a breed-specific genomic marker that can serve as a practical tool for verifying breed purity, thereby supporting the conservation of this valuable indigenous genetic resource.

## Simple Summary

The Leizhou black goat, a local breed from southern China, is famous for its excellent meat. However, its small size limits how much meat it can produce. Breeders often cross it with larger Nubian goats to get bigger, faster-growing offspring, but it is not well understood which specific genes are responsible for this improvement or if it affects meat quality. In this study, we compared the entire genetic blueprint of purebred and crossbred goats to find these key genes. Our analysis discovered several genes that are likely important for muscle growth and are part of a system linking nerve signals to muscle development. We also found a unique genetic variation that is common in Nubian goats and many other breeds worldwide but is completely missing in purebred Leizhou goats. This makes it a perfect genetic “fingerprint” to tell if a goat is a pure Leizhou breed. These findings help us understand the biology behind improved growth in crossbred goats and provide breeders with concrete genetic targets and a simple test to aid in conserving and improving this valuable goat breed.

## 1. Introduction

Growth performance and skeletal muscle development are pivotal economic traits in meat goat production, serving as primary determinants of productivity and profitability. As typical quantitative traits, their manifestation is predominantly governed by complex polygenic systems [1,2]. The Leizhou black goat, an indigenous breed in southern China, is highly valued for its exceptional adaptability to hot and humid climates, robust disease resistance, and superior meat quality. However, its relatively small body frame (average adult weights of approximately 50 kg for bucks and 43 kg for does) and a conventional dressing percentage ranging from 42% to 46% constrain its ultimate meat yield and production efficiency [3].

Understanding the genetic basis of growth differences between purebred and crossbred populations is essential for designing effective conservation and breeding strategies. Crossbreeding with high-performance exotic breeds, such as the Nubian goat, has been employed as a means to enhance growth performance in local breeds [4]. Empirical evidence indicates that crossing Leizhou black goats with Nubian bucks yields F1 progeny exhibiting superior early-growth vigor, with 8-month-old crossbred male kids demonstrating significantly higher body weight compared to purebred Leizhou kids [5]. This phenotypic gain underscores the substantial genetic potential unlocked through hybridization. In parallel, the advent of genomic technologies has facilitated the dissection of the molecular underpinnings of these traits. Preliminary investigations utilizing transcriptomic sequencing and copy number variation (CNV) analyses have identified several candidate genes implicated in myogenesis and growth regulation within the Leizhou black goat genome, offering valuable initial clues [6,7]. Despite these advances, a significant knowledge gap persists. The comprehensive genetic architecture underlying the phenotypic shifts in crossbred populations, which include accelerated growth and increased stature, potentially alongside alterations in meat quality attributes, remains unresolved [8]. More critically, a systematic, genome-wide comparative analysis aimed at identifying highly differentiated genomic regions, key genetic variants, and regulatory networks specifically associated with differences in muscle development between purebred and crossbred Leizhou black goats is still lacking.

Herein, we performed comparative whole-genome resequencing of purebred Leizhou black goats and their Nubian-crossbred counterparts. Our objectives were threefold: first, to evaluate genome-wide genetic diversity and population structure between the two groups; second, to uncover genomic regions exhibiting elevated genetic differentiation through population differentiation analysis; and third, to screen and annotate candidate genes and genetic variants within these regions that may be associated with phenotypic divergence in growth and muscle development. The resulting findings offer meaningful genomic insights and molecular resources that advance our understanding of the genetic regulation of muscle development in goats. These outputs are expected to support the conservation of this valuable indigenous genetic resource and inform future molecular breeding efforts.

## 2. Materials and Methods

### 2.1. Sample Collection and Library Construction

Blood samples were collected from two distinct populations of Leizhou black goats. The purebred Leizhou black goat (WL) group (*n* = 22) was sourced from the Leizhou Black Goat Conservation Farm in Danzhou City, Hainan Province. The crossbred group (JN) (*n* = 50), consisting of later-generation crossbreds derived from crossing Leizhou black goats with Nubian bucks as sires, was obtained from the Hainan Dongfang Jinnong Black Goat Breeding Co., Ltd., (Dongfang, China). All animals were maintained under standardized feeding and nutritional management conditions to minimize environmental variance. Approximately 5 mL of venous blood was aseptically drawn from the jugular vein of each animal using vacuum tubes pre-treated with EDTA-K_2_ as an anticoagulant. The collected blood samples were immediately placed on dry ice, transported to the laboratory, and stored at −80 °C for subsequent genomic DNA extraction.

Genomic DNA was isolated from the whole blood samples using a standard phenol–chloroform protocol [9]. The concentration and purity (A260/A280 and A260/A230 ratios) of the extracted DNA were quantified using a Nanodrop 2000 spectrophotometer (Thermo Fisher Scientific, Waltham, MA, USA). DNA integrity was assessed by 1% agarose gel electrophoresis. Only samples with a total DNA yield exceeding 1500 ng and showing no signs of degradation were selected for subsequent library preparation. A total of 72 qualified DNA samples (22 WL and 50 JN) were used for whole-genome resequencing. Sequencing libraries were constructed following the manufacturer’s instructions, and paired-end (150 bp) sequencing was performed on an Illumina HiSeq 2000 platform by Wekemo Tech (Shenzhen, China) Co., Ltd.

### 2.2. Variation Calling and Annotation

Raw sequencing reads were first assessed using FastQC (v0.11.9) for exploratory visualization of base quality scores and sequence content. Adapter contamination and low-quality bases were then removed using Trimmomatic (v0.39) [10] with fixed, pre-set parameters. These trimming parameters were not adjusted based on the FastQC results. The high-quality clean reads were aligned to the goat reference genome (ARS1.2, Assembly GCF_001704415.2) using the BWA-MEM (v0.7.17) algorithm [11]. SAMtools (v1.9) was used to sort the alignment files and mark PCR duplicates [12]. The overall mapping rate and average sequencing depth for each sample were calculated using Qualimap (v2.2.3) [13].

Single-nucleotide polymorphisms (SNPs) and small insertions/deletions (InDels) were identified using the Genome Analysis Toolkit (GATK, v4.1.9.0) best practices pipeline [14]. Briefly, HaplotypeCaller in GVCF mode was used for each sample, followed by joint genotyping of all samples using GenotypeGVCFs. The raw variant call set was subjected to a multi-step filtering process to ensure high-confidence variants: (1) Hard-filtering was applied using GATK’s VariantFiltration with the criteria: QD < 2.0 || MQ < 40.0 || FS > 60.0 || SOR > 3.0 || MQRankSum < −12.5 || ReadPosRankSum < −8.0; (2) Bcftools (v1.22) was used to retain only biallelic sites [12]; and (3) PLINK (v1.9.0) was employed for further filtering based on genotype missing rate (<10%), minor allele frequency (>0.05), and Hardy–Weinberg equilibrium (*p* > 0.001) [15].

### 2.3. Population Genetic and Highly Differentiated Genomic Region Analysis

Principal Component Analysis (PCA) was performed based on the genome-wide SNP dataset using PLINK (v1.9.0) to visualize the genetic relationship and population structure between the purebred and crossbred groups. The genetic differentiation between the two populations (WL vs. JN) was evaluated by calculating the population differentiation index (*F*_ST_) for each SNP using VCFtools (v0.1.16) [16]. Genomic regions exhibiting elevated genetic differentiation were identified as those containing SNPs with *F*_ST_ values in the top 0.01% of the empirical distribution. Genes located within or adjacent to these genomic windows were extracted as candidate selected genes. The GGVD database (http://animal.omics.pro/code/index.php/GoatVar, last accessed: 19 August 2025) was utilized to further validate the distribution of SNPs within these potential highly differentiated genomic regions across different breed populations [17].

### 2.4. Functional Annotation and Enrichment Analysis

The functional consequences of the filtered SNPs were annotated using SnpEff (v5.2.1) with the Capra hircusARS1.2 genome annotation database [18]. To explore the biological functions of the candidate genes identified from the highly differentiated genomic regions analysis, Gene Ontology (GO) and Kyoto Encyclopedia of Genes and Genomes (KEGG) pathway enrichment analyses were performed using the DAVID online bioinformatics resource (v6.8) [19]. Terms with a Benjamini–Hochberg corrected *p*-value (FDR) < 0.05 were considered significantly enriched. The RGD database (http://animal.omics.pro/code/index.php/RGD, last accessed: 12 June 2026) was utilized to examine the tissue expression profiles of the potential highly differentiated genes [20].

## 3. Results

### 3.1. Sequencing Data Quality and Population Structure

Whole-genome resequencing of 22 purebred (WL) and 50 crossbred (JN) Leizhou black goats generated approximately 2233.2 GB of raw data. After stringent quality control, the high-quality clean reads were aligned to the goat reference genome (ARS1.2). The average sequencing depth was 12.68× for the WL group and 12.69× for the JN group, with both groups achieving an average mapping rate exceeding 96.8% (Supplementary Table S1), indicating high-quality data suitable for subsequent variant analysis.

**Figure 1:**
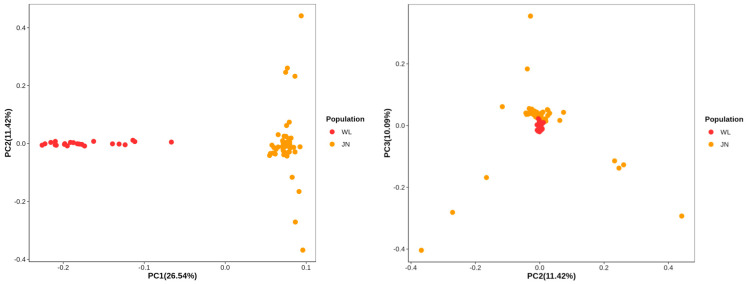
Principal component analysis of purebred and crossbred Leizhou Black goats. Different colors represent different populations, with red indicating purebred Leizhou Black goats (WL) and orange indicating crossbred Leizhou Black goats (JN).

Principal component analysis (PCA) based on genome-wide SNPs was performed to assess the population genetic structure. The first three principal components were analyzed, collectively accounting for 48.05% of the total genetic variation (PC1: 26.54%, PC2: 11.42%, PC3: 10.09%). PC1 alone distinctly separated the two populations, with the purebred WL group clustered predominantly at negative values (PC1 < 0) and the crossbred JN group at positive values (PC1 > 0), indicating a clear primary axis of genetic differentiation. While PC2 and PC3 provided auxiliary resolution, they exhibited overlapping distributions. Specifically, the WL population centered around zero in both PC2 and PC3, whereas the JN population displayed a broader distribution spanning both positive and negative values (Figure 1). This multi-dimensional clustering pattern confirms a significant genetic structure difference between the WL and JN groups, providing a reliable foundation for subsequent comparative genomic analysis.

### 3.2. Genome-Wide SNP Detection and Functional Annotation

After joint genotyping and stringent filtering, a high-confidence set of SNPs was obtained for subsequent analyses. Functional annotation of these SNPs using SnpEff revealed that the majority were located in non-coding regions, which is consistent with general patterns in mammalian genomes. Specifically, 55.885% of SNPs were found in introns and 32.815% in intergenic regions (Table 1). In contrast, only 0.236% of SNPs were located in exonic regions. Among the functionally important variant types identified were 2136 frameshift variants, 51 stop-gained variants, and 3188 splice region variants (Table 2).

### 3.3. Identification of Selection Signals and Candidate Genes

**Figure 2:**
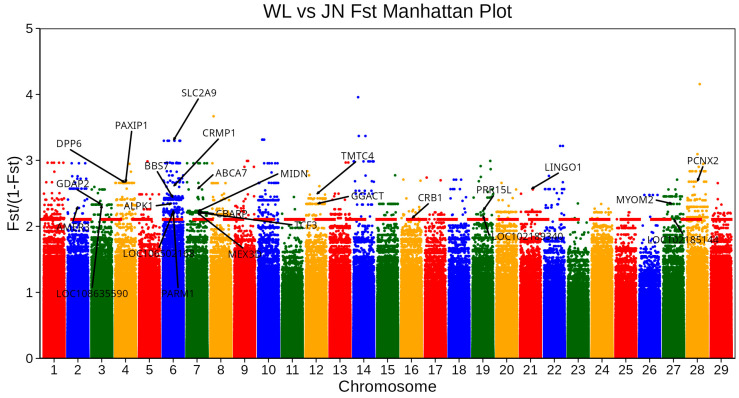
Genome-wide *F*_ST_ Manhattan plot between purebred (WL) and crossbred (JN) populations of Leizhou Black goats. Each point represents an SNP. The *x*-axis indicates chromosome position, and the *y*-axis shows the *F*_ST_/(1 − *F*_ST_) value. The red dashed line represents the significance threshold (top 0.01%). SNPs above the threshold indicate genomic regions with strong genetic differentiation between the two populations.

To identify genomic regions that may have diverged during the crossbreeding process, we calculated the *F*_ST_ between the WL and JN groups across the genome. The Manhattan plot of *F*_ST_ values revealed several prominent peaks, indicating regions with strong genetic divergence (Figure 2). Genomic windows containing SNPs in the top 0.01% of the *F*_ST_ distribution were defined as candidate highly differentiated genomic regions (Supplementary Table S2).

Genome-wide screening for highly differentiated genomic regions, visualized in the *F*_ST_ Manhattan plot (Figure 2), revealed several prominent peaks exceeding the empirical significance threshold (top 0.01%) across multiple autosomal chromosomes. Notably, the most significant peaks were identified on chromosomes 6, 9, 14, and 19. These highly differentiated regions, characterized by extreme *F*_ST_ values, were defined as candidate highly differentiated genomic regions. A number of genes were located within or in proximity to these high-*F*_ST_ loci. Key candidate genes identified include *TMTC4*, *LINGO1* and *PRR15L*, and *MYOM2*. The specific positioning of these genes within the significant peaks strongly suggests their involvement in the phenotypic divergence between the two populations. These genes are functionally associated with critical biological processes relevant to growth and muscling traits, such as glucose metabolism and transport (*SLC2A9*), transmembrane signaling (*TMTC4*), neuronal development (*LINGO1*), and cytoskeletal organization (*MYOM2*).

### 3.4. Functional Enrichment Analysis of Candidate Genes

**Figure 3:**
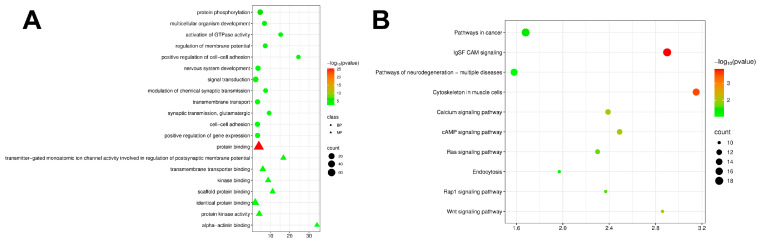
GO (**A**) & KEGG (**B**) Enrichment Analysis of Candidate Genes.

GO analysis revealed significant enrichment in terms related to molecular functions and biological processes crucial for muscle and tissue development. The most significantly enriched molecular function was “protein binding” (79 genes, P = 4.53 × 10^−26^). Key enriched biological processes included “protein phosphorylation”, “multicellular organism development”, “signal transduction”, and “positive regulation of cell–cell adhesion” (Figure 3A). Enrichment for terms like “nervous system development” and “modulation of chemical synaptic transmission” was also observed.

KEGG pathway analysis further highlighted specific signaling pathways involved in muscle biology. Significantly enriched pathways included “Cytoskeleton in muscle cells”, “cAMP signaling pathway”, “Calcium signaling pathway”, “Ras signaling pathway”, and “Wnt signaling pathway” (Figure 3B). The enrichment of the “IgSF CAM signaling” pathway also underscores the potential role of cell adhesion mechanisms. More detailed results of the GO and KEGG functional enrichment analysis are provided in Supplementary Table S3.

### 3.5. Distribution Analysis of Key SNPs in Selected Regions

Furthermore, we examined the distribution of SNPs within the identified highly differentiated genomic regions and discovered two loci that exhibited completely distinct allele frequencies between the purebred WL and crossbred JN populations. Specifically, the mutations rs646826802 (RNGTT: NC_030816.1:g.48446934C>T; intron variant) and rs6558652193 (NC_030836.1:g.24688596G>T) were absent in all purebred WL individuals but were present in heterozygous or homozygous mutant states in the crossbred descendants (Supplementary Table S4).

**Figure 4:**
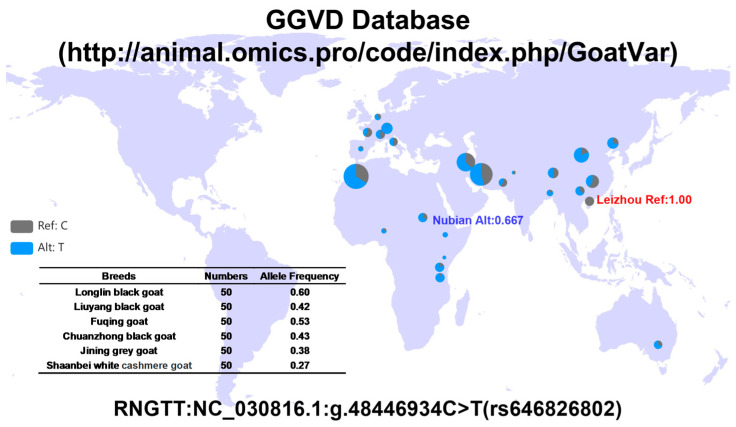
Global Allele Frequency Distribution of the Selected SNP (rs646826802) in the Goat GGVD Database. Distribution of the alternate allele (T) for the RNGTT gene (rs646826802) across various goat breeds worldwide, as retrieved from the GGVD database. The blue circles represent breeds where the mutation was detected. The table below lists the specific allele frequencies for the five analyzed Chinese indigenous breeds.

The mutant allele (T) of rs646826802 was found at a high frequency (0.667) in the Nubian goat population, which served as the paternal line for the crossbred group. This distinct pattern prompted further investigation. Comparative analysis using global goat SNP distribution data from the GGVD database revealed that rs646826802 was undetectable only in the Leizhou black goat, while it was present in all 25 other worldwide breeds examined (Figure 4). To validate this finding, we designed specific primers for this locus and performed Sanger sequencing on DNA samples from 50 individuals each of six additional Chinese indigenous goat breeds, utilizing previously collected laboratory resources (Supplementary Table S5). The sequencing results confirmed the presence of this mutation in all other tested breeds, highlighting its unique absence in the purebred Leizhou black goat.

## 4. Discussion

This study delineates the comprehensive genetic landscape differentiating purebred Leizhou black goats from their Nubian-crossbred counterparts, revealing substantial genomic divergence that may underlie their distinct growth and muscularity phenotypes. The reliability of our dataset for identifying highly differentiated genomic regions is supported by high-quality sequencing data and a clear population structure. Notably, the crossbred JN population exhibited a broader distribution in the PCA plot compared to the tightly clustered purebred WL population. This dispersion reflects the genetic complexity of the JN group, which consists of later-generation crossbreds with variable proportions of Nubian ancestry and incomplete admixture fixation. Consequently, the distinct genetic clustering observed here reflects the significant genomic impact of introducing Nubian ancestry, which correlates with the enhanced growth performance of the crossbred animals. These findings suggest a functional genomic framework that may contribute to these phenotypic outcomes. The identified genes and variants constitute valuable candidate markers for subsequent research and breeding applications.

Consistent with mammalian genomic architecture, the vast majority of detected SNPs were located in non-coding regions. However, the identification of functional variants, including frameshift and stop-gained mutations within coding sequences, suggests plausible mechanistic avenues for phenotypic modulation. The core of our analytical insights stems from the highly differentiated genomic regions, which pointed to several high-priority candidate genes. Among them, *CBARP*, *LINGO1*, and *MYOM2* stand out for their well-established connections to muscle biology. CBARP (also known as C19orf26) encodes a calcineurin inhibitor that is predominantly expressed in fast-twitch skeletal muscle in mice, where it likely modulates calcium-dependent signaling involved in fiber-type specification and metabolic adaptation [21]. MYOM2 (myomesin-2) is a structural component of the sarcomeric M-band, essential for myofibrillar assembly and muscle contraction. In goats, it has been linked to meat quality [22], and its expression differs between pigs with varying growth rates [23], as well as across developmental stages in poultry [24,25]. LINGO1 is best known as a neuronal protein that negatively regulates oligodendrocyte differentiation; in a muscle context, its selection could influence motor neuron function or neuromuscular junction stability, thereby indirectly affecting muscle innervation and maintenance. Supporting this idea, *LINGO1* expression varies among pig breeds with different meat quality traits [26] and has also been associated with Wooden Breast myopathy in broilers [27]. In addition, several other loci identified in this study, such as *PAXIP1* [28], *ABCA7* [29], *CRMP1* [30], *DPP6* [31], and *MIDN* [32], have been implicated in neuronal development and signal transduction in humans and mice. Taken together, these observations support a model of coordinated selection acting on an integrated regulatory network that connects neural input with muscle function. Although the functional evidence for several of these genes derives primarily from studies in humans, mice, or other model organisms, their conserved roles across vertebrates lend biological plausibility to their involvement in caprine muscle development. The concurrent selection of genes directly governing muscle structure and those modulating its neural regulation may have acted synergistically, ultimately contributing to the enhanced growth and muscularity observed in the crossbred population [33,34]. While direct experimental validation in goats is needed to confirm these hypotheses, the convergence of genomic signals onto a neuromuscular regulatory framework provides a compelling starting point for future functional studies.

GO and KEGG enrichment analyses collectively present a coherent picture of the biological processes and pathways under putative selection. The significant enrichment in terms such as “protein phosphorylation” and “signal transduction,” along with specific pathways including cAMP, calcium, Ras, and Wnt signaling, is highly indicative of their central role. These pathways are established regulators of satellite cell activation, myoblast proliferation and differentiation, and overall muscle metabolism [35,36,37].

The strong signal for the “Cytoskeleton in muscle cells” pathway directly aligns with the structural functions of MYOM2, highlighting convergent selection on genes governing the physical architecture of muscle tissue. Furthermore, the enrichment of terms related to “cell–cell adhesion” and the “IgSF CAM signaling” pathway points to selective pressures affecting fundamental processes like myoblast fusion and tissue organization, which are crucial for muscle growth and repair [38,39]. An intriguing finding is the enrichment of terms associated with nervous system development and synaptic transmission. This suggests that the genetic basis for improved growth and muscularity is not solely attributable to selection on genes expressed within the muscle fibers themselves. Instead, it likely involves coordinated selection acting on the integrated neuromuscular system. To further support the biological plausibility of the candidate genes identified in this study, we examined their tissue expression profiles using the RGD database. Eight genes, including *MYOM2*, *CBARP*, and *DPP6*, showed detectable expression in muscle or nerve tissues, consistent with their proposed roles in neuromuscular signaling and muscle development. Notably, *LINGO1* and *MIDN* exhibited relatively high expression levels across multiple muscle and brain tissues, further strengthening their potential involvement in the neural regulation of muscle function (Supplementary Table S6).

Furthermore, the distinct global distribution pattern of the RNGTT gene mutation (rs646826802) provides compelling evidence for its utility as a molecular marker. The complete absence of the mutant allele (T) in the purebred Leizhou black goat, contrasted with its high frequency (0.667) in the paternal Nubian breed and its common presence in numerous other global breeds, establishes a clear genetic signature. This stark contrast in allele frequency between the Leizhou black goat and other breeds, including its direct crossbreeding parent, highlights a unique genetic feature of the purebred population. It should be noted that this is a non-coding intronic variant, and its functional relevance to growth or muscle development remains unknown. Nevertheless, its distinct allele frequency distribution makes this locus a highly informative indicator for breed identification. The specific genotype at rs646826802 can effectively distinguish purebred Leizhou black goats from crossbred individuals and from most other goat breeds, offering a practical genetic tool for verifying breed purity in conservation and breeding programs.

This study has certain limitations. A primary constraint is the limited sample size of the purebred Leizhou black goat group (*n* = 22), a consequence of the challenging real-world conservation context. Over the past two decades, extensive genetic admixture with other breeds has occurred, and as a result, genetically verified purebred individuals in southern China are now almost exclusively confined to conservation farms, with a total population of fewer than 200 animals. This restricted accessibility may affect the statistical power and bias allele frequency estimations. Future studies would benefit from validating these findings in larger cohorts or employing analytical methods that account for uneven sample sizes. Another methodological consideration is the average sequencing depth (~12.7×), which is somewhat lower than the 15–20× depth commonly recommended for accurate population-level SNP genotyping. Although we applied stringent quality filters to minimize the impact of genotype uncertainty, moderate sequencing depth may introduce residual noise in allele frequency estimation and *F_ST_* calculation. Nonetheless, because our identification of highly differentiated regions relies on genome-wide empirical distributions rather than absolute *F_ST_* thresholds, random genotyping errors are unlikely to systematically drive the extreme differentiation signals observed at the top-ranked loci. Still, future studies employing deeper sequencing or imputation-based approaches would help refine the resolution of candidate regions. We further performed a post hoc power analysis to evaluate the detection capacity of our study design. Given the sample sizes of WL (*n* = 22) and JN (*n* = 50), the minimum detectable true pairwise *F_ST_* at α = 0.05 and 80% power was estimated to be 0.08. This theoretical threshold accounts for sampling error alone; considering additional genotyping uncertainty due to moderate sequencing depth, genetic differentiation weaker than this value cannot be reliably identified in our dataset. However, the top 0.01% extreme *F_ST_* outliers examined in this study exhibit values substantially exceeding this threshold, confirming that our core findings focus on regions of strong and robust population divergence. In the present study, we performed preliminary validation of the population distribution for specific key loci, which supported the reliability of our core findings. However, the current conclusions primarily rely on genomic associations and in silico functional predictions.

Furthermore, it should be noted that without a pure Nubian reference population or admixture analysis, the high-*F*_ST_ signals observed here cannot be unequivocally attributed to selection for growth traits, as they may partly reflect breed-of-origin differences. Therefore, these candidate genes should be interpreted as suggestive targets warranting further functional validation and association testing. Additionally, the proposed involvement of these genes in a neuromuscular regulatory network, while supported by known functional annotations from the literature, remains a hypothesis derived from genomic data rather than an experimentally validated mechanism. Direct evidence for altered neural signaling, neuromuscular junction function, or muscle fiber composition would require targeted functional studies, such as gene expression profiling, electrophysiological measurements, or histological analyses, which were beyond the scope of the current work. To substantiate these links, subsequent work should integrate gene expression profiling to correlate genotypes with transcriptional activity and employ functional assays to confirm the causal roles of key mutations. Furthermore, integrating metabolomic or proteomic data could connect these genetic variants to downstream physiological changes, providing a more complete understanding of the trade-offs and synergies between growth and carcass traits. In line with this direction, we are currently pursuing single-cell transcriptomic, ATAC-seq, and metabolomic analyses in Leizhou black goats to achieve a more precise and comprehensive understanding of the regulatory networks governing muscle development.

## 5. Conclusions

This study used whole-genome resequencing to investigate the genetic basis for improved growth in crossbred Leizhou black goats. Analysis identified candidate genes within a neuromuscular regulatory network, offering potential clues to the genetic factors that may contribute to heterosis. Notably, the *RNGTT* (rs646826802) allele was absent in purebred goats but common in the paternal Nubian line and globally, establishing it as a specific molecular marker for breed authentication. These findings provide a functional understanding of muscle-related genetic divergence and deliver a practical tool for verifying breed purity, a critical step for the conservation and sustainable utilization of this valuable indigenous genetic resource.

## Figures and Tables

**Table 1:** Distribution and Proportion of SNPs across Different Genomic Regions.

Variants Types	Number	Rate
DOWNSTREAM	138,157	4.866%
EXON	6707	0.236%
GENE	32	0.001%
INTERGENIC	931,639	32.815%
INTRON	1,586,590	55.885%
SPLICE_SITE_ACCEPTOR	254	0.009%
SPLICE_SITE_DONOR	43	0.002%
SPLICE_SITE_REGION	2871	0.101%
TRANSCRIPT	13,270	0.467%
UPSTREAM	128,674	4.532%
UTR_3_PRIME	26,429	0.931%
UTR_5_PRIME	4378	0.154%

**Table 2:** Functional Impact Categories and Distribution of SNPs.

Variants Types	Number	Rate
3_prime_UTR_variant	26,429	0.930%
5_prime_UTR_variant	4386	0.154%
bidirectional_gene_fusion	31	0.001%
conservative_inframe_deletion	382	0.013%
conservative_inframe_insertion	347	0.012%
disruptive_inframe_deletion	740	0.026%
disruptive_inframe_insertion	308	0.011%
downstream_gene_variant	138,160	4.860%
frameshift_variant	2136	0.075%
gene_fusion	1	0.000%
intergenic_region	931,639	32.774%
intragenic_variant	13,200	0.464%
intron_variant	1,589,590	55.920%
non_coding_transcript_exon_variant	2852	0.100%
non_coding_transcript_variant	70	0.002%
splice_acceptor_variant	277	0.010%
splice_donor_variant	104	0.004%
splice_region_variant	3188	0.112%
start_lost	9	0.000%
stop_gained	51	0.002%
stop_lost	20	0.001%
stop_retained_variant	7	0.000%
upstream_gene_variant	128,674	4.527%

## Data Availability

The data that support the findings of this study are available on Figshare database (https://doi.org/10.6084/m9.figshare.32399187, 25 May 2026).

## Supplementary Materials

The following supporting information can be downloaded at: https://www.mdpi.com/article/10.3390/biology15131038/s1, Table S1: Summary statistics of whole-genome resequencing data quality; Table S2: WL_vs_JN.weir.valid.sorted.type.top0.01%; Table S3: KEGG and GO for JN WL DEGs; Table S4: Population distribution for JN WL SNPs; Table S5: Frequency Distribution of the rs646826802 in six Goat breeds; Table S6: Multi-tissue expression profiles of candidate genes.
